# A current perspective on cancer immune therapy: step-by-step approach to constructing the magic bullet

**DOI:** 10.1186/s40169-016-0130-5

**Published:** 2017-01-03

**Authors:** Gabriele D’Errico, Heather L. Machado, Bruno Sainz

**Affiliations:** 1 Department of Biochemistry, School of Medicine, Autónoma University of Madrid, Calle del Arzobispo Morcillo 4, 28029 Madrid, Spain; 2 Department of Biochemistry and Molecular Biology, Tulane University School of Medicine, 1430 Tulane Ave, #8543, New Orleans, LA 70112 USA; 3 Department of Cancer Biology, Instituto de Investigaciones Biomédicas “Alberto Sols” CSIC-UAM, Madrid, Spain; 4 Enfermedades Crónicas y Cáncer Area, Instituto Ramón y Cajal de Investigación Sanitaria (IRYCIS), Madrid, Spain

**Keywords:** CTLA-4, PD-1, PD-L1, Immunotherapy, Coley’s toxin

## Abstract

Immunotherapy is the new trend in cancer treatment due to the selectivity, long lasting effects, and demonstrated improved overall survival and tolerance, when compared to patients treated with conventional chemotherapy. Despite these positive results, immunotherapy is still far from becoming the perfect magic bullet to fight cancer, largely due to the facts that immunotherapy is not effective in all patients nor in all cancer types. How and when will immunotherapy overcome these hurdles? In this review we take a step back to walk side by side with the pioneers of immunotherapy in order to understand what steps need to be taken today to make immunotherapy effective across all cancers. While early scientists, such as Coley, elicited an unselective but effective response against cancer, the search for selectivity pushed immunotherapy to the side in favor of drugs focused on targeting cancer cells. Fortunately, the modern era would revive the importance of the immune system in battling cancer by releasing the brakes or checkpoints (anti-CTLA-4 and anti-PD-1/PD-L1) that have been holding the immune system at bay. However, there are still many hurdles to overcome before immunotherapy becomes a universal cancer therapy. For example, we discuss how the redundant and complex nature of the immune system can impede tumor elimination by teeter tottering between different polarization states: one eliciting anti-cancer effects while the other promoting cancer growth and invasion. In addition, we highlight the incapacity of the immune system to choose between a fight or repair action with respect to tumor growth. Finally we combine these concepts to present a new way to think about the immune system and immune tolerance, by introducing two new metaphors, the “push the accelerator” and “repair the car” metaphors, to explain the current limitations associated with cancer immunotherapy.

## Introduction

Since war was declared on cancer in 1971, our arsenal of drugs against this enemy has steadily increased. The first class of drugs developed, conventional chemotherapy [1], provided significant benefits for the treatment and management of different cancers; however, conventional chemotherapy has two intrinsic defects: lack of selectivity [2] and long-term resistance [3]. The subsequent development of targeted therapies, including monoclonal antibody-based therapies, such as rituximab, overcame the lack of selectivity associated with conventional chemotherapy by targeting specific proteins involved in cancer cell stimulation, proliferation or apoptosis evasion [4]. While monoclonal antibody-based strategies have significantly increased in clinical practice over the past decade, mechanisms of acquired resistance remains a hurtle, likely due to genetic and epigenetic instability of cancer cells [5]. Thus, despite significant advances made in cancer treatment over the past 45 years, we are still far from developing therapies capable of effectively ablating cancer while avoiding adverse effects on healthy cells and loss of efficacy over time.

Starting with Coley’s toxin, followed by Paul Ehrlich’s hypothesis of tumor surveillance and contrasted by Burnet’s immunological tolerance theory [6], the idea that the immune system can play a pro- and/or anti-tumor role has been recognized and debated for years. As the immune system is armed to protect against pathogens, it has been long postulated that immune cells should recognize tumor cells as foreign, and effectively eliminate them before spreading to distant organs. This concept, today referred to as cancer immunotherapy, has the potential to be the magic bullet that investigators have been desperately searching for since the early 1900s [7].

The last century was a pendulum, swinging between big hope and deep disappointment in the immunotherapy field. Why has our view of immunotherapy shifted from promising to disappointing? Can immunotherapy be made more effective? In this review we look to the past to understand the current problems associated with immunotherapy. We present a new way to think about the immune system and immune tolerance. In addition to the standard “fuel the engine, release the brake” rules of immunotherapy, we introduce the “push the accelerator” and “repair the car” metaphors to explain part of the current limitations associated with cancer immunotherapy.

### Immunotherapy: a revolutionary view of cancer treatment

Immunotherapy was born in 1890. Its father, William Coley [8], observed that a patient with an inoperable sarcoma that suffered a *Streptococcus pyogenes* infection twice obtained complete remission. Based on this observation, Coley treated approximately 1000 patients with inoperable cancers (specially sarcomas) with a mixture consisting of killed *S. pyogenes* and *Serratia marcescens*, achieving a complete remission in 10% of treated patients. Compared with actual 5-year survival rates for metastatic sarcoma (20%) [9], the effects of Coley’s toxin were promising and demonstrated that our immune system could effectively eradicate tumors with a low rate of adverse effects in a subset of patients. While the mechanism of action of Coley’s toxin was unknown at the time, Coley’s toxin essentially activated tumor-infiltrating leukocytes [heterogeneous populations of cells, including varying proportions of neutrophils, macrophages, T and B cells, and natural killer cells (NK)]. Following administration of Coley’s toxin, dendritic cells (DCs), professional antigen presenting cells (APCs), initiate an immune response by presenting the captured bacterial antigen to naïve CD4^+^ or CD8^+^ T cells [10] in lymphoid tissues, which results in T cell priming [11] and inflammatory interleukin production [IL-1, IL-2, tumor necrosis factor alpha (TNFα,) IL-12] [12, 13]. Clonal T cell expansion triggers a humoral immune response by activating B cells, or cellular immunity by activating Th1 effector cells or NK cells [14]. Thus, as tumors often display a high degree of leukocyte infiltration, it is reasonable that stimulation of tumor-infiltrating leukocytes can result in tumor cell targeting and elimination.

### The first gauntlet: release the brake

In 1949, some years after Coley’s first experiments, Macfarlane Burnet stated, “if in embryonic life expendable cells from a genetically distinct race are implanted and established, no antibody response should develop against the foreign cell antigen when the animal takes on independent existence” [6, 15]. Peter Medawar would go one step further and propose that the immune system becomes tolerant to cancer cells due to the similarities that exist with normal healthy cells. In his early experiments, Medawar injected embryonic mouse donor cells into mice of a different strain, rendering the recipient mice tolerant to future grafts from the donor and not third-party strains [16]. These experiments set the initial groundwork for what would become the concept of acquired immunological tolerance. Even if the molecular mechanisms explaining Medawar and Burnet’s observations would not be discovered until 25 years later, Medawar and Burnet had already thrown down the first gauntlet to modern immunotherapy [16].

Immunological tolerance is a fundamental process, the lack of which would result in numerous pathologies including autoimmune illnesses. Since Burnet and Medawar won the Nobel Prize for their pioneering work in this field more than 50 years ago, an enormous amount of progress has been made to better understand immune tolerance and the numerous redundant mechanisms involved in this biological process. In 1959, Joshua Lederberg published nine propositions on immunity and tolerance [17]. The sixth proposition stated that “the immature antibody-forming cell is hypersensitive to an antigen–antibody combination: it will be suppressed if it encounters the homologous antigen at this time”, highlighting that each antibody-producing cell has a single specificity [17]. In 1978, Nossal and Pike experimentally demonstrated that bone marrow-derived cells cultured with an antigen became tolerant to the antigen in a time-dependent manner, with maximal tolerance being achieved only when the antigen was present continuously as the cultured bone marrow cells matured [18]. In the late 1980’s, Kappler demonstrated central tolerance in mice and indicated that tolerance induction may occur in the thymus [19]. Goodnow later showed that B cells that reactive to “self” antigens are eliminated or silenced in order to avoid autoimmunity [20]. And finally, Le Douarin demonstrated that the thymus is not only important for central tolerance but it produces cells [i.e. regulatory T (T_reg_) cells] that strongly regulate effector cells, discovering a third dominant form of immune tolerance [21].

Taken together these results clearly indicated that self-immune tolerance is maintained and regulated by multiple mechanisms, including similarity with self-antigens, regulatory immune cells, a suppressive versus activating cytokine balance and immune checkpoints. Thus, if this process is to be effectively manipulated in order to release the brake, we have to stimulate APCs with an antigen significantly different from “self” to recruit effector cells, balance cytokines to obtain a pro-inflammatory milieu, and finally, inhibit immune checkpoints to avoid tolerance.

### Hope dies last

The idea of immune tolerance put forth by Medawar and Burnet dampened the hope generated by Coley’s experiments that activating the immune system could treat cancer. However, in 1953, Foley demonstrated that methylcholanthrene-induced tumor cells could produce immunogenic antigens in mice, although strain-dependent differences were observed [22]. Nathrath would add additional fuel to the fire by showing that the host immune system is capable of recognizing new molecular properties (antigens) displayed by tumor cell as a result of the changes accumulating during the transition from normal to malignant cells [23]. Interestingly, however, the greatest hope for immunotherapy would come from another illness described in the 1950s, autoimmune diseases such as lupus, which clearly confirmed that auto-reactive cells can elude self tolerance [24]. Finally, towards the later end of the 20th century, immunogenic tumor associated antigens were discovered in mice [25] and humans [26], resulting from cancer cell genomic instability. Unsettling still was the fact that while cancer cells could express a multitude of new and unknown antigens as a result of malignant transformation, the immune system was not stimulated to target these antigens/cells. The problem therefore was no longer the absence of immunogenic antigens on cancer cells, but rather understanding why our immune system “ignores/tolerates” cells harboring these antigens.

In the absence of inflammation, naïve T-cells circulate preferentially to secondary lymphoid tissue [27]. During an infection, APCs respond to inflammatory cytokines (IL-1, TNF-α) [28] and migrate via afferent lymph vessels to lymph nodes where they can interact with naïve T-cells. In a similar manner, macrophage or B cells can take up and process free antigen in the blood or spleen [29]. The interaction of T cell CD28 and DC CD80 (B7-1) or CD86 (B7-2) allows for specific T cells to proliferate in the paracortex [30] and become competent to receive further activation signals from antigen-bearing macrophages and B cells. This process results in the production of cytokines, loss of L-selectin (which is involved in lymph node entry) and increased expression of adhesion molecules like VLA 4 (which facilitates extravasation into non-lymphoid tissue) [31]. Macrophages and parenchymal cells produce inflammatory cytokines (IL-1, TNF-α) that increase expression of selectins and integrin ligands. Ultimately, activated T-cells express adhesion molecules that allow them to selectively enter inflamed tissues expressing counterpart adhesion molecules. In summary, immune cells can present antigens (dendritic cells, macrophages), produce cytokines (macrophage) and interact with cells of the adaptive immune system (B-cells, T-cells) in the lymph nodes, resulting in their subsequent activation via receptor/ligand interactions. Once activated, adaptive immune cells proliferate, alter their receptors and adhesion molecules, and finally migrate to initiate the destruction of foreign pathogens.

To avoid indiscriminate activation and self-destruction, the immune system has developed redundant mechanisms to tolerate self or non-dangerous antigens. Since central tolerance is not the underlying mechanisms by which cancer cells escape immune targeting, we refer the reader to several published reviews detailing the biology of central tolerance (Ref. [32, 33]). Peripheral tolerance, on the other hand, is the primary mechanism utilized by cancer cells to avoid the immune system. In the early 1990s, Jenkins and Schwartz demonstrated that T-cells need a co-stimulatory signal to fully activate, and if T-cells receive only TCR signals they become anergic [34]. The co-stimulatory signals must be received from APCs [35]: B7-1 (CD80) or B7-2 (CD86) on APCs binding to CD28 on T-cells is necessary to fully stimulate T-cells. While an important step forward in our understanding of T-cell regulation, the big discovery was not B7-1 or B7-2, but rather the receptor that inhibits the second co-stimulatory signal. James Allison, director of the UC Berkeley Cancer Research Laboratory, was intrigued with a molecule called cytotoxic T-lymphocyte antigen-4 (CTLA-4) [36], originally discovered in a cDNA library derived from activated T-cells. In the late 1990s, Allison and his group began to study how CTLA-4 inhibits T-cells and if this inhibition could explain why T-cells do not attack cancer cells. They demonstrated that CTLA-4, a homologue of CD28, bound with higher affinity (at least 10-fold) to both B7-1 and B7-2 [37] and inhibited CD4^+^ T-cell activation. Under certain conditions, T-cells up-regulate CTLA-4, which binds to B7-1 and B7-2 with a higher affinity than CD28, effectively hijacking the second co-stimulatory signal that T-cells require for full T-cell activation, proliferation, and effector function [38]. As a result, T-cells cannot be fully activated and thus undergo anergy. Since its discovery, several groups have worked diligently towards dissecting the role of CTLA-4. Studies with CTLA-4^−/−^ mice confirmed its inhibitory function in vivo. Waterhouse et al. showed that mice lacking CTLA-4 died early on of fatal lymphoproliferative disorders [39], demonstrating that CTLA-4 acts as a negative regulator of T cell activation and is vital for the control of lymphocyte homeostasis. Based on ever increasing data demonstrating a role for CTLA-4 as a negative regulator of T-cell activation, Allison and colleagues went on to show that in vivo administration of antibodies to CTLA-4 promoted the rejection of tumors, including pre-established tumors, confirming that CTLA-4 blockage can allow for, and potentiate, an effective immune responses against tumors [40].

### The first victory in the new era

These studies and others [41–43] suggested that monoclonal antibody-mediated CTLA-4 blockage could represent an effective anti-cancer therapy. To translate these findings to the clinical setting, the Medarex Corporation generated a series of monoclonal antibodies using a unique transgenic mouse (HuMAb), in which the endogenous murine immunoglobulin genes had been knocked out and replaced with the human loci [44]. Ipilimumab showed safety in a phase I study and efficacy in a phase III study (ClinicalTrials.gov Identifier: NCT00094653) with primary overall survival endpoints. Patients allocated to receive ipilimumab had a median overall survival of 10.1 versus 6.4 months for the control group [hazard ratio (HR), 0.68; *P* ≤ 0.003]. Based on the promising results, Bristol-Meyer’s Yervoy^®^ (ipilimumab) was approved by the US Food and Drug Administration (FDA) and the European Medicines Agency (EMA) in 2011 for the treatment of metastatic melanoma [38].

While CTLA-4 was the first “checkpoint” inhibitor identified, the list of checkpoint immunomodulators continues to grow, with inhibitors of the programmed cell death protein 1/ligand pathway leading the way. Programmed cell death protein 1, also known as PD-1 and CD279 (cluster of differentiation 279) is a member of the CD28 superfamily expressed on activated CD4^+^ and CD8^+^ T-cells as well as NK and B-cells while programmed death-ligand 1 (PD-L1), also known as cluster of differentiation 274 (CD274) or B7 homolog 1 (B7-H1), is expressed predominantly on APCs. The main role of PD-1 is to act like a stopwatch to limit the activity of T-cells in the “battle field” during the effector phase of T-cell activation in peripheral tissues and the tumor microenvironment via the delivery of negative signals upon interaction with its two ligands (PD-L1 or PD-L2). PD-L1 and PD-L2 compete for PD-1 [45] and upon binding both inhibit T cell proliferation, cytokine production and cell adhesion [46], although some contradictory data have suggested a costimulatory function [47]. T-cells begin to express PD-1 when activated and its expression increase over time [48, 49], while PD-L1 is expressed on APCs present in the inflamed tissue. PD-1 is also highly expressed on T_reg_ cells, where it may enhance their proliferation in the presence of ligand [50]. The expression on T_reg_ cells also highlights the role PD-1 plays in regulating the induction and maintenance of peripheral tolerance and protection from autoimmune attack [51]. Our current understanding of the PD-1/PD-L1 pathway shows that engagement of PD-1 and PD-L1 leads to inactivation of effector T-cell molecules such as Zap70 to inhibit T-cell proliferation, thus limiting the inflammatory damage in inflamed tissues [52]. For example, during chronic infections the PD-1/PD-L1 pathway leads to anergy [53]. It appears as though several cancers, including lung, ovarian and colon carcinomas as well as melanomas, have evolved to over express PD-L1 [54]. Thus, chronic tumor associated antigen exposure in cancer can lead to high levels of PD-1 expression on T-cells, which can interact with PD-L1 expressed on cancer cells, inhibiting T-cell activation and perhaps inducing a state of anergy in immune cells present in the tumor. Blocking the PD-1/PD-L1 pathway can revert this condition, promoting cancer cell elimination by activated T-cells. In the past 5 years, Bristol-Myers Squibb has produced a fully humanized antibody against PD-1 named Opdivo (Nivolumab), which obtained FDA accelerated approval in 2014 [55] based on the “Study of Nivolumab (BMS-936558) Compared With Dacarbazine in Untreated, Unresectable, or Metastatic Melanoma”.

Today many check-point inhibitors are used by oncologist to achieve significant increases in survival rates (e.g. 1- and 2-year survival rates of 62 and 43%, respectively for melanoma or 1- and 2-year survival rates of 42 and 23%, respectively for lung cancer) and/or to achieve a durable partial or complete response in cancer patients (e.g. 31% for melanoma patients) [56, 57]. Table 1 summarizes the current FDA- and EMA-approved immune checkpoint inhibitors. Clinical studies have also investigated the efficacy of combination therapies using anti-PD-1/PD-L1 therapies together with other checkpoint inhibitors, such as the anti-CTLA4 treatment ipilimumab. The combination of nivolumab and ipilimumab increased overall survival in patients with untreated melanoma. The median progression-free survival was 11.5 months (95% confidence interval [CI], 8.9–16.7) with nivolumab plus ipilimumab, as compared with 2.9 months (95% CI, 2.8–3.4) with ipilimumab (hazard ratio for death or disease progression, 0.42; 99.5% CI, 0.31–0.57; P < 0.001), and 6.9 months (95% CI, 4.3–9.5) with nivolumab (hazard ratio for the comparison with ipilimumab, 0.57; 99.5% CI, 0.43–0.76; P < 0.001) [58]. It is important to stress that even if combination therapy allows us to obtain better results and improved medium overall survival, it is still far from becoming the perfect therapy. The current underlying problem with immune checkpoint inhibitors is that if there are no T-cells in the tumor border then there are no effector cells capable of eliminating the tumor cells. Therefore, even if we release the brake we cannot obtain clinically relevant results. Thus, the next strategy lies in “fueling the engine”, that is allowing T-cells to reach the tumor border.

**Table 1:** Summary of immune therapies in clinical use

Immune therapy	Target	Stage	Cancer type	Ref.
Ipilimumab	CTLA-4	Clinical use	Advanced melanoma	107
Nivolumab	PD-1	Clinical use	Melanoma Renal cancer NSLC	108 109 110
Pembrolizumab	PD-1	Clinical use	Melanoma	111
Atezolizumab	PD-L1	Clinical use	NSLC Clear renal cancer bladder cancer	112 113
Sipileucel	Peripheral blood mononuclear cells	Clinical use	Prostate cancer	114

### Fueling the engine

As stated above, immune checkpoints inhibitors are only effective if tumors are infiltrated with T-cells. Therefore, if a tumor lacks infiltrated T-cells, immune checkpoint inhibitors are essentially ineffective. Cancer vaccines have been extensively investigated as a strategy to induce T-cell infiltration in the tumor—“fuel the engine”. Currently, however, Sipileucel is the only “cellular immunotherapy” (i.e. vaccine therapy) approved by the FDA. Sipileucel consists of autologous peripheral blood mononuclear cells (PBMCs), obtained by leukapheresis and cultured with a prostatic acid phosphatase linked to granulocyte–macrophage colony-stimulating factor (GM-CSF) [59]. Results from the 9902B study in patients with prostate cancer demonstrated an overall survival of 25.8 months for patients receiving Sipileucel compared to 21.7 months for patients who received the control treatment [59].

While Sipileucel demonstrates the clear benefit of “fueling the engine”, the use of cancer vaccines in other tumor types is still in the experimental stages. For example, Kleponis et al. developed a GM-CSF-secreting pancreatic cancer vaccine (GVAX) that provided maturation signals to APCs at the local vaccine site. Stimulated APCs processed tumor antigens and presented them to T-effector cells, allowing T-cells to infiltrate the tumor [60]. The authors went on to show that PD-L1 expression was induced in the infiltrating cells (T-cells). This study demonstrated that vaccine-based therapies may have adequately primed pancreatic cancer for anti-PD-1/PD-L1 treatments, highlighting that this typically T-cell-effector poor/T_reg_-rich tumor [61] could be potentially treated with PD-1/PD-L1 inhibitors. Thus, cancer vaccine-based immunotherapy may overcome the resistance of certain cancers to immune checkpoint inhibitors, while immune checkpoint inhibitors may enhance the efficacy of the cancer-vaccine therapies. The goal of a combination strategy is to combine the strength of each immunotherapy approach, with cancer vaccines functioning to “fuel the engine” and immune checkpoint inhibitors working to “release the brake”.

Perplexing is the fact that PD-1 and CTLA-4 checkpoint inhibitors, even when helped by cancer vaccines, are not effective against all cancer types, nor do they work in every patient with the same cancer. Perhaps other immune cell types are negatively affecting cancer immunotherapy? The explanation we put forward to explain this dilemma is that in some cancers we have the machinery (the car) on a downhill slope, so if we release the brake (immune checkpoint inhibitors) the car can move. In contrast, when we are on an uphill slope or on a plain field, releasing the brake simply does not move the car. For such scenarios, we have to release the brake and push the accelerator.

Looking deep inside the immune system we can find a dynamic and complex environment of cells that are different in type, size, complexity, markers and function. Even the same cell can exist in two (or more) different polarized states. For example, macrophages can switch between a pro-inflammatory (classically activated) and a reparative (alternatively activated) state [62]. T-cells can be stimulated into T effector cells or T_regs_ cells, each of which can have a very distinct role within a tumor [63]. Thus, we need to take a step back and understand why, when and how a cell (i.e. macrophage, T-cell, etc.) can switch from a classically activated (inflammatory macrophage or T effector cell) to an alternatively activated (pro-tumorigenic macrophage or T_reg_) state and what mediates this change. Answering these questions may provide the key to push the accelerator.

### Immune cells: Dr. Jekyll and Mr. Hide

The immune system is comprised of many cells, including but not limited to dendritic cells, mast cells, macrophages, neutrophils and lymphocytes. In addition, and to complicate the matter even more, immune cells are extremely plastic and each cell type can differentiate into at least two forms depending on their environment and the paracrine signals they receive (reviewed in [64–66]). Lymphocytes can differentiate into many subsets. Apart from B and T cells, T lymphocytes can differentiate into CD8^+^ or CD4^+^ T cells, the latter of which can in turn differentiate into T_reg_ and T_helper_ cells [67]. DCs that present captured antigens to naïve T-cells, have two major subsets: myeloid (i.e. conventional DC or immunogenic DC) and plasmacytoid form (or tolerogenic DC) [68]. As described above, macrophages can exist in at least two forms [69], and in the context of a tumor, macrophages and neutrophils can differentiate into tumor-associated macrophages (TAMs) or neutrophils (TANs) [70], respectively.

**Fig. 1:**
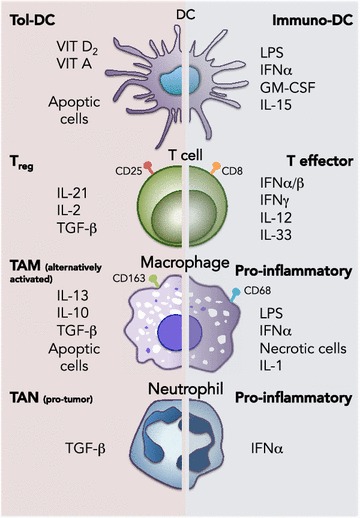
The two faces of immune cells: The Dr. Jekyll and Mr. Hide concept. Immune cells, including dendritic cells (DCs), T-cells, macrophages and neutrophils, are extremely plastic and can assume different roles/functions depending on factors encountered at the site of infection or within the tumor microenvironment. When stimulated by factors such as TGF-ß or when in contact with apoptotic cells, immune cells become pro-tumorigenic (*left side*) and differentiate/polarize towards tolerogenic DCs (Tol-DC), T_reg_ cells, tumor-associated macrophages (TAMs) or tumor-associated neutrophils (TANs). In contrast, when stimulated by pro-inflammatory cytokines, such as IFNs, IL-1 or IL-12, immune cells become anti-tumorigenic (*right side*) and differentiate/polarize towards immunogenic DCs (immuno-DC), T effector cells, activated pro-inflammatory macrophages or neutrophils

The interest in the different states of immune cells, particularly within the tumor, stems from the different biological affects these cells produce depending on their state, polarization or differentiation (Fig. 1). For example, human DCs can exist as immunogenic or tolerogenic DCs [71], with immunogenic DCs functioning primarily to stimulate a T_helper_ [72] response while tolerogenic DCs function primarily to stimulate a T_reg_ response [73]. Moreover, the ratio of different DCs depends on the cytokine milieu. In vitro studies clearly demonstrate that GM-CSF, interferon alpha (IFNα), or IL-15 can induce the differentiation of inflammatory DCs while IL-10, vitamin A or D3, or immunosuppressive drugs such as cyclosporine A induce tolerogenic DCs through E-cadherin mediated signaling [74, 75]. Thus, depending on the cytokine milieu, DCs can elicit a strong immune response or a tolerogenic state.

Macrophages represent another important cell type that play a pivotal role in activating and shaping the immune response, and similar to DCs, a dichotomy has been proposed for macrophage activation: classically or alternatively activated [76, 77]. IFNα, LPS or inflammatory cytokines can induce classical activation while IL-4, IL-13, TGF-β and reparatory signals can induce alternative activation of macrophages. In the last decade, if macrophages have gone from a negligible player in tumor progression to a pivotal modulator of tumor growth and metastasis, it is due to the identification of TAMs. There is now solid and continuously growing evidence to show that TAMs actively promote all aspects of tumor growth and development including promotion of angiogenesis, matrix remodeling and suppression of adaptive immunity [78]. Recent studies also show that TAMs share many characteristics with alternatively activated macrophages such as (1) activation of the arginase pathway, implicated in arginine metabolism; (2) promotion of cell repair and proliferation in strong opposition with the NOS pathway that promote cells killing [79]; (3) production of IL-10 and vascular endothelial growth factor (VEGF) over other factors promoting cell survival [80]; (4) production of matrix metalloproteinases (MMPs), implicated in cancer initiation and metastasis [81, 82]; (5) activation of NFκB and STAT3 signaling, enhancing tumor progression by directly communicating with cancer stem cells (CSCs) [62], and (6) activation of STAT6, which possess potent inhibitory activity of T-cell activity [83].

T-cells, the soldiers of the immune system, have a fundamental role in immune surveillance, and even for these cells a dichotomy exists: a T-cell can defend the host from cancer while aiding tumor growth. Zhang et al. showed that ovarian carcinoma patients with high tumor infiltrating lymphocytes had improved 5-year survival rates compared to patients with low tumor infiltrating lymphocytes [84]; however, in other cancers, such as renal cancer, high tumor infiltrating lymphocytes translated into worse prognosis [85]. These clear opposing observations are now explained by differences in the type of infiltrating T-cells: T_reg_ versus T effectors cells (TCD8^+^). T_reg_ cells, a subgroup of T-cells expressing CD4 and CD25, regulate activation of other T-cells and are necessary to maintain peripheral tolerance to self-antigens. We have now come to understand that increased numbers of T_reg_ cells present in the tumor can have a negative prognostic impact. For example, Sato et al. showed that a high TCD8^+^/T_reg_ cell ratio translated into better overall survival while the opposite was seen for a high T_reg_/TCD8^+^ ratio [86]. Likewise, Li et al. demonstrated that the efficiency and percent depletion of T_reg_ cells from a tumor can improve cancer outcome [67, 87]. Specifically, they show that in Foxp3.LuciDTR-4 mice, which show 90–95% T_reg_ depletion, large established tumors completely regressed, unlike anti-CD25 antibody-mediated T_reg_ elimination, which is less efficient (approximately 70%). Thus, high-level depletion of T_reg_ cells is necessary for tumor regression.

Like a coin, immune cells have two faces, one with a strong potential to fight cancer and the other (the opposite one) strongly promoting cancer development and immune system escape. This Dr. Jekyll and Mr. Hide concept has been re-coined the “corrupted policemen” concept by Bonavita et al. in order to stress the fact that those cells born to protect the host can become corrupt and turn against it to favor cancer growth [88]. This switch is complex and partly mediated by cytokines: inflammatory cytokines promoting immune system cells to show their anti-cancer face while anti-inflammatory cytokines promote the pro-cancer side to dominate. But cytokines are only mediators. More important are the stimuli that induce immune host cells to convert into either cancer allies (pro-tumor macrophages, T_reg_ cells, or tolerogenic DC) or cancer enemies (inflammatory macrophage, CD8^+^ T-cells, T_helper_ cells or immunogenic DC) (Fig. 1). If we discover which mechanism(s) induce the inflammatory anti-cancer response and which ones induce the pro-cancer response, in theory we could pharmacologically change the face of the coin, favoring an anti-cancer response.

### To fight or to repair? That is the question

The first problem that oncoimmunotherapists faced was immune tolerance. Currently, a more challenging dilemma lies in the ability of the immune system to balance itself between two opposing actions: “fight” the enemy or “repair the damage”. A successful immune response can be accompanied by extensive tissue damage [89]. Fortunately, the immune system has the capacity to repair the resulting damage via T_reg_ cells, macrophages and anti-inflammatory cytokines that send a repair message to the site of damage. In the context of cancer, however, the wrong choice can have detrimental effects on tumor eradication, as alluded to above. Numerous studies have shown that tumor growth and even post treatment tumor cell death can be perceived by the immune system as a repair signal, initiating a wound healing response that can favor sustained tumor growth and even tumor chemoresistance via immune cell secreted factors (reviewed in [90, 91]). Dissecting how the immune system senses and responds to damage will improve our efforts of inhibiting the immune system from favoring tumor growth over tumor destruction.

After immune system activation, the battlefield is replete with damaged cells, the majority being apoptotic cells (e.g. bacteria, neutrophils, epithelial cells). It is therefore logical to think that these cells could regulate the immune system or represent the signal that promotes a repair response. Although conventional and targeted therapies often aim to induce apoptosis, these strategies may themselves be carcinogenic [92].

The relation between cancer, immune cells and dead/apoptotic cells has gained increased attention over the past decade. Apoptosis obtained through activation of caspases or mitochondrial chain dysfunction was historically declared as non-immunogenic, while necrotic death accompanied by the release of proteins, lipids and other cellular debris from cells has been widely considered as strongly immunogenic [93, 94]. Chemotherapy-mediated cell death was accepted as an apoptosis-mediated process, inducing an immunosuppressive milieu of cytokines [95], but studies show that depending on the agent used and the degree of cell death induced, chemotherapy can also induce necrotic cells [96]. Moreover, other studies have shown that classically-induced apoptotic cells can profoundly affect the immune system [97], and the idea that chemotherapy-induced apoptosis is non-immunogenic may be overstated [98–100].

In cancer, there appears to be a ying-yang scenario with respect to the presence of dead cells and method of cell death induction. The presence of apoptotic cells can be sensed and translated into a “need to repair” action, allowing T_reg_/Th2 stimulation through cytokine expression (IL-10, IL-13) [100, 101]. On the other hand, necrotic cells or highly variable tumor associated antigens can be sensed and translated into a “need to fight” action, allowing T-CD4^+^ stimulation thought inflammatory cytokine expression (TNF-α, IL-1, etc.) [93]. Williams et al. showed that DCs exposed to apoptotic Jurkat cells or apoptotic primary T-cells failed to maturate and were unable to support CD4^+^ allogeneic T-cell proliferation, as compared to DCs exposed to lipopolysaccharide (LPS) or necrotic cells [102]. Conversely, phosphatidylserine exposed on apoptotic epithelial cells suppressed IFN-β production by dendritic cells via inhibitory signalling mediated by the cell-surface glycoprotein CD300a and thus suppressed T_reg_ cell proliferation [103]. Moreover, Kleinclauss et al. in 2006 demonstrated that apoptotic cells induce CD4^+^ T-cells to express CD25^+^ (a marker of T_reg_ cells) inducing a state of tolerance [104]. Taken together, these studies demonstrate that the immune system can sense the type of danger and activate a specific action. In our laboratory we have discovered that apoptotic pancreatic tumor cells, as opposed to necrotic or live cells, can strongly induce immune system suppression or a pro-Th2 state, promoting repair signals that favor pancreatic tumor growth and chemoresistance (unpublished data). Our observations are in line with data published by Wu et al. where they show that intravenous administration of donor apoptotic splenocytes promotes the generation of tolerogenic DCs and the expansion of T_reg_ cells in the pancreas; in vivo clearance of either DCs or T_reg_ cells abrogated immune tolerance induction [105]. Thus, in pancreatic cancer where chemotherapies such as gemcitabine induce tumor cell apoptosis, an immune response that favors tumor growth may be activated.

## Conclusion

The field of cancer immunotherapy has been driven by scientists such as William Coley whose important results set the foundation for modern day immunotherapy, as well as by scientists who while opposed the concept of immunotherapy made pinnacle discoveries that favored the evolution of immunotherapy into one of the most promising techniques in place today to battle cancer. The scientific community is convinced that immunotherapy is only a step away from becoming the magic bullet to defeat cancer. While the future looks promising, there are still many hurdles that need to be overcome.

We now have the capacity to “fuel the engine” by using vaccine strategies to accumulate effector T-cells at the tumor border, and “release the brake” to allow T-cells to fight the cancer by inhibiting immune system checkpoints. This strategy allows us to fight cancer when the battlefield is downhill or when the tumor entity itself is “immunogenic” due to (1) profound differences in tumor associated antigens between normal cells and tumor cells or (2) the presence of immunogenic necrotic cells. This strategy, however, is ineffective when the battlefield is plain or uphill, or when the tumor itself promotes a “need to repair” response due to chemotherapy-induced apoptosis or other unknown factors. Thus, when faced with this scenario, an approach to ensure that the immune system senses the cancer as danger in order to promote a “need to fight” over a “need to repair” response is essential. Consequently, the “push the accelerator” component of the universal magic bullet is still lacking. This action may possibly be achieved by educating APCs (DCs and macrophages) to sense every cancer as “dangerous”, ensuring an anti-tumor immune response in all cases. Moreover, understanding that certain immune cells (e.g. macrophages) can shift the immune response in one direction over another, combination therapies may be necessary to transiently eliminate other immune cells. Ultimately, the goal is to unmask those factors that promote a “need to repair” response while at the same time enhancing those factors that are sensed as a danger signal by the immune system. Only then will immunotherapy truly become the magic bullet for cancer treatment.

## Abbreviations

DC: dendritic cells
APC: antigen presenting cells
IL: interleukin
NK: natural killer
T_reg_: T-regulatory cell
CTLA-4: cytotoxic T-Lymphocyte antigen-4
PD-1: programmed cell death protein 1
PD-L1: programmed death-ligand 1
PBMC: peripheral blood mononuclear cells
IFN: interferon
TNF: tumor necrosis factor
GM-CSF: granulocyte–macrophage colony-stimulating factor
TAM: tumor-associated macrophage
TAN: tumor-associated neutrophil
